# Nanocarrier-based targeted drug delivery for Alzheimer’s disease: addressing neuroinflammation and enhancing clinical translation

**DOI:** 10.3389/fphar.2025.1591438

**Published:** 2025-05-14

**Authors:** Kang Wang, Rongying Yang, Jing Li, Haitao Wang, Li Wan, Jiale He

**Affiliations:** ^1^ Acupuncture and Moxibustion Department, Beijing Massage Hospital, Beijing, China; ^2^ Key Laboratory of Medical Electrophysiology, Ministry of Education & Medical Electrophysiological Key Laboratory of Sichuan Province, (Collaborative Innovation Center for Prevention of Cardiovascular Diseases), Institute of Cardiovascular Research, Southwest Medical University, Luzhou, Sichuan, China; ^3^ Department of Radiology, Shengjing Hospital of China Medical University, Shenyang, Liaoning, China; ^4^ The school of Clinical Medical Sciences, Southwest Medical University, Luzhou, Sichuan, China; ^5^ Department of Neurosurgery, The First Hospital of China Medical University, Shenyang, Liaoning, China; ^6^ Department of Rheumatology, Guang’anmen Hospital, China Academy of Chinese Medical Sciences, Beijing, China

**Keywords:** Alzheimer’s disease, nanocarriers, neuroinflammation, blood-brain barrier, drug delivery, nanotechnology, clinical translation

## Abstract

Alzheimer’s disease (AD) is a progressive neurodegenerative disorder characterized by cognitive decline, amyloid-beta (Aβ) aggregation, tau pathology, and chronic neuroinflammation. Among these, neuroinflammation plays a crucial role in exacerbating disease progression, making it an attractive therapeutic target. However, the presence of the blood-brain barrier (BBB) significantly limits the effective delivery of therapeutic agents to the brain, necessitating novel drug delivery strategies. Nanocarrier-based delivery systems have emerged as a promising solution to these challenges, offering targeted drug transport, enhanced BBB penetration, and improved bioavailability while minimizing systemic toxicity. This review explores the current advancements in nanocarrier-mediated drug delivery for AD, focusing on the mechanisms of neuroinflammation, the role of nanocarriers in overcoming the BBB, and their ability to modulate inflammatory pathways. Furthermore, the review discusses preclinical validation strategies and key challenges, including safety concerns, large-scale production limitations, and regulatory hurdles that must be addressed to enable clinical translation. Future perspectives emphasize the integration of nanotechnology with precision medicine, gene therapy, and artificial intelligence to optimize nanocarrier design for individualized AD treatment. By overcoming these obstacles, nanocarriers hold the potential to revolutionize therapeutic approaches for AD and other neurodegenerative diseases.

## 1 Introduction

Alzheimer’s disease (AD), a progressive neurodegenerative disorder (Scheltens et al., 2021), remains one of the leading causes of dementia worldwide, affecting millions of individuals and placing an immense socioeconomic burden on healthcare systems (Li et al., 2022). It is characterized by progressive cognitive decline, memory loss, and personality changes, which are ultimately fatal (Scheltens et al., 2021). Recent global estimates suggest that there are approximately 416 million individuals across the continuum of AD, including those in the early stages such as preclinical and prodromal AD. Specifically, 32 million individuals are affected by AD dementia, 69 million by prodromal AD, and 315 million by preclinical AD, with these groups constituting 22% of all persons aged 50 and above (Gustavsson et al., 2023). The underlying pathophysiology of AD is complex, involving the accumulation of amyloid-beta plaques, hyperphosphorylation of tau proteins, and the disruption of neural networks (Yang Y. et al., 2022; Taylor et al., 2023; Ashrafian et al., 2021; Otero-Garcia et al., 2022). Despite extensive research, effective disease-modifying treatments remain elusive, with current therapies primarily aimed at symptomatic relief rather than halting disease progression (Lista et al., 2023; Companys-Alemany et al., 2022). Table 1 summarizes the drugs approved by the FDA for the treatment of AD.

**TABLE 1:** FDA-approved drugs for the treatment of Alzheimer’s disease.

Drug name	Type	Mechanism
Donepezil (Birks and Harvey, 2018)	Cholinesterase Inhibitor	Increases acetylcholine levels in the brain
Rivastigmine (Birks and Grimley Evans, 2015)	Cholinesterase Inhibitor	Inhibits acetylcholinesterase and butyrylcholinesterase
Galantamine (Lim et al., 2024)	Cholinesterase Inhibitor	Inhibits acetylcholinesterase and modulates nicotinic receptors
Memantine (Matsunaga et al., 2018)	NMDA Receptor Antagonist	Regulates glutamate activity to prevent excitotoxicity
Lecanemab (van Dyck et al., 2023)	Amyloid Beta Antibody	Targets and clears amyloid plaques in the brain
Aducanumab (Kim et al., 2024)	Amyloid Beta Antibody	Reduces amyloid plaques by targeting and removing them

Several risk factors have been identified in the development and progression of AD. These include genetic factors such as the presence of the apolipoprotein E (APOE) ε4 allele, which is strongly associated with an increased risk of AD (Raulin et al., 2022). Environmental factors, including head trauma, cardiovascular diseases, and diabetes, have also been implicated in elevating the risk of developing AD (Brett et al., 2022; Khemka et al., 2023). Additionally, lifestyle factors such as physical inactivity, poor diet, and low educational attainment have been shown to contribute to a higher susceptibility to AD (Khemka et al., 2023). Among the various contributing factors to AD pathology, neuroinflammation has emerged as a critical player. Neuroinflammation is marked by the activation of microglia and astrocytes in response to neuronal injury, which, while protective in the early stages, can exacerbate neuronal damage and contribute to disease progression when chronic (Wang et al., 2023; Al-Ghraiybah et al., 2022). The release of pro-inflammatory cytokines, such as tumor necrosis factor-alpha (TNF-α), interleukin-1β (IL-1β), and IL-6 (Ng et al., 2018), along with the generation of reactive oxygen species (ROS) (Park et al., 2021), creates a microenvironment that accelerates neurodegeneration. Recent studies have shown that targeting neuroinflammation could potentially halt or slow down the neurodegenerative process, making it an attractive therapeutic strategy for AD (Calsolaro and Edison, 2016; Mangalmurti and Lukens, 2022).

**FIGURE 1:**
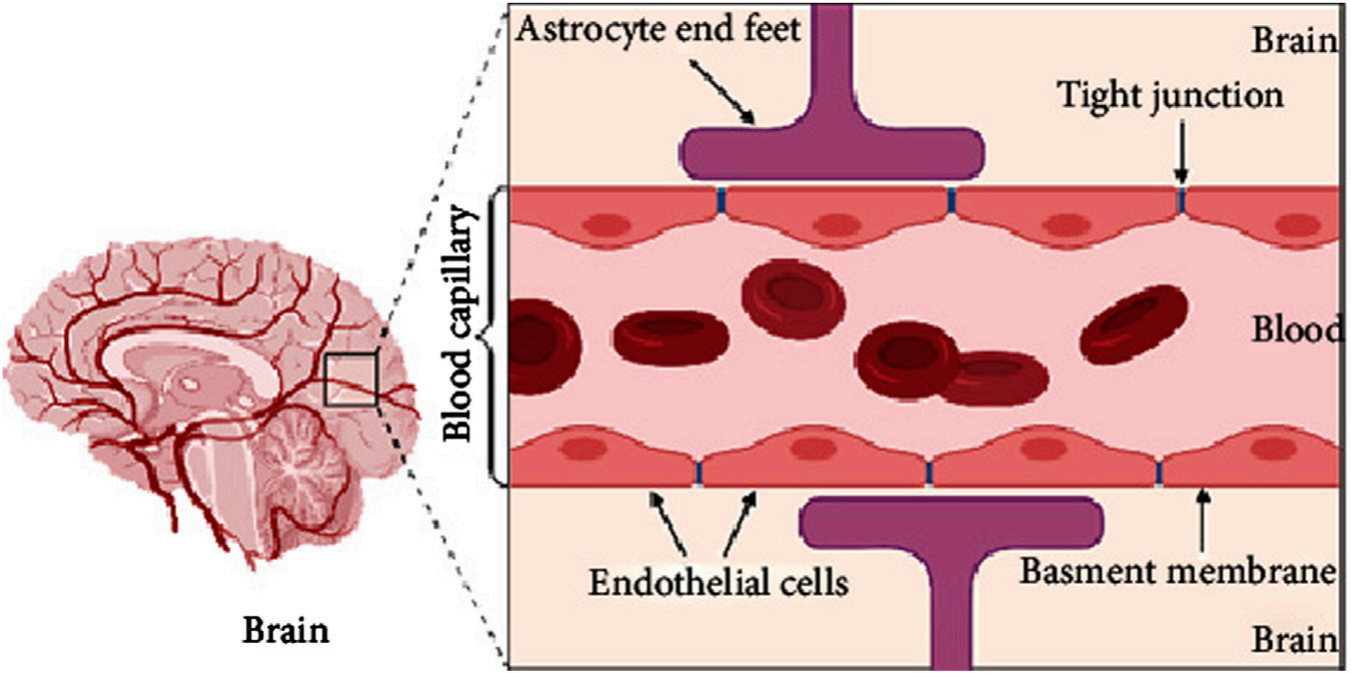
A schematic diagram of brain and simple longitudinal zoom in blood brain barrier (Created by BioRender). Extracted from author Abeer Alahmari (Alahmari, 2021). Copyright © 2021 Abeer Alahmari.

One of the major obstacles in developing effective treatments for AD is the blood-brain barrier (BBB). The BBB serves as a selective barrier that protects the brain from potentially harmful substances, but it also significantly impedes the delivery of therapeutic agents (Wong et al., 2019), including those aimed at modulating neuroinflammation. Traditional drug delivery methods, such as oral or intravenous administration, often fail to deliver sufficient concentrations of drugs to the brain, limiting their therapeutic potential (Cassano et al., 2021). Therefore, overcoming the BBB remains a central challenge in AD drug development. Figure 1 provides a schematic diagram illustrating the structure of the BBB (Alahmari, 2021).

In recent years, nanotechnology has provided innovative solutions to this challenge. Nanocarriers, including liposomes, polymeric nanoparticles, and dendrimers, offer a promising platform for the targeted delivery of drugs to the brain (Zhou et al., 2018; Saeedi et al., 2019). Their small size, surface modifiability, and ability to encapsulate a wide variety of therapeutic agents make them ideal candidates for crossing the BBB and precisely targeting areas of neuroinflammation (Yang X. et al., 2022). Additionally, nanocarriers can enhance the pharmacokinetics and bioavailability of drugs, while minimizing systemic toxicity (Wen et al., 2023; Cai et al., 2023). This ability to deliver small molecules directly to inflamed neural tissues could significantly improve therapeutic outcomes in AD, especially in relation to neuroinflammation-driven mechanisms.

Despite these promising advancements, there remain significant challenges in translating nanocarrier-based drug delivery systems from preclinical to clinical settings. The precise targeting of nanocarriers to specific regions of the brain, ensuring their stability in the bloodstream, and managing potential immune responses to the nanomaterials are critical issues that need to be addressed. Furthermore, the integration of nanotechnology with other treatment strategies, such as gene therapy or immunomodulation, could provide synergistic benefits and lead to more effective treatments for AD.

This review aims to explore the multifaceted role of nanocarriers in targeting neuroinflammation in AD. We will discuss the current state of nanotechnology for drug delivery to the brain, focusing on how nanocarriers can be designed to cross the BBB, target neuroinflammatory pathways, and improve therapeutic efficacy. We will also examine the preclinical and clinical challenges associated with these systems and propose future directions for research to overcome these barriers.

## 2 Neuroinflammation and Alzheimer’s disease

Neuroinflammation has long been recognized as a hallmark of AD pathology. It is a complex, multifactorial process that involves the activation of resident immune cells in the brain, particularly microglia, as well as astrocytes (Leng and Edison, 2021). Under normal conditions, microglia act as the primary defenders of the central nervous system (CNS), responding to damage and maintaining homeostasis through phagocytosis and the release of pro-inflammatory cytokines (McNamara et al., 2023). However, in AD, this protective response becomes dysregulated, and chronic activation of microglia and astrocytes is observed, exacerbating neuronal damage and promoting the progression of neurodegeneration (Jorfi et al., 2023; Wu and Eisel, 2023).

**FIGURE 2:**
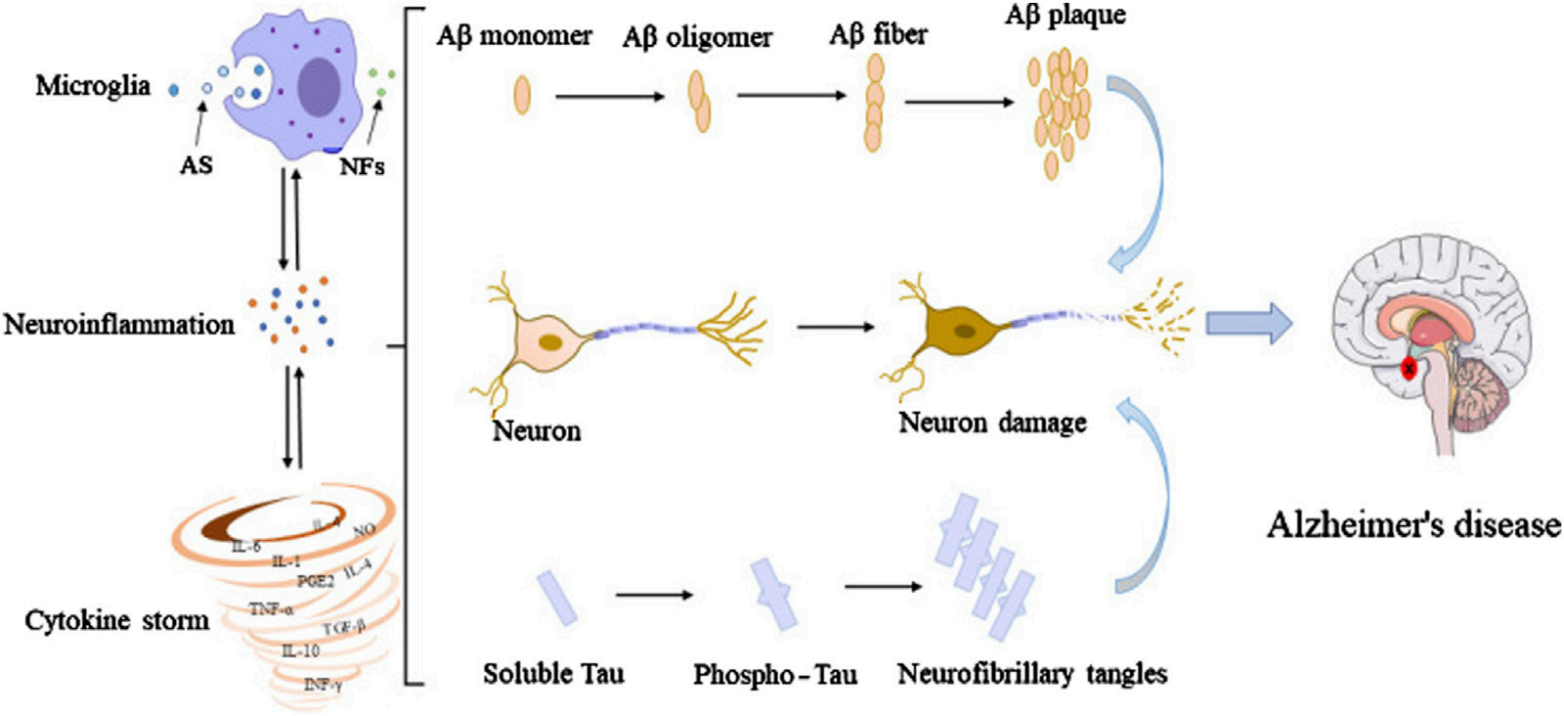
Interconnections among microglia, Aβ, tau, and neurons. In their early developmental stages, microglia enhance the phagocytic capacity and generate phagocytoma abnormal material, such as cell debris and misfolded proteins. Subsequently, M2 microglial cells are promoted to release proinflammatory mediators, leading to an inflammatory storm. In this process, the amyloid monomers gradually accumulated to form amyloid plaques. Meanwhile, free tau proteins are phosphorylated and aggregated in neurons to form neurofibrillary tangles. The accumulation of Aβ plaques and the formation of neurofibrillary tangles could positively feedback to microglia to promote the activation of microglia, leading to impaired ability to phagosome foreign objects. Extracted from author Wang et al. (Wang et al., 2023). Copyright © 2023 Wang, Zong, Cui, Wang, Wu, Wang, Liu and Lu.

The activation of microglia is one of the earliest responses to neuronal damage in AD. Microglia detect signals from damaged or dying neurons and, in turn, become activated. This activation is marked by changes in their morphology and the release of inflammatory mediators such as pro-inflammatory cytokines, chemokines, and ROS (Johnson et al., 2020; Mary et al., 2024). In particular, the cytokines TNF-α, IL-1β, and IL-6 have been implicated in driving the inflammatory response in AD (Singh, 2022). Elevated levels of these cytokines are commonly found in the brains of AD patients and contribute to the neurotoxic environment that accelerates neuronal injury (Lopez-Rodriguez et al., 2021). Furthermore, microglia-mediated inflammation interacts with amyloid-beta (Aβ) plaques, a pathological hallmark of AD, by promoting their formation and exacerbating their toxic effects (Gerrits et al., 2021). Figure 2 illustrates the interconnections among microglia, Aβ, tau, and neurons, highlighting how microglial activation leads to an inflammatory response that contributes to the accumulation of Aβ plaques and neurofibrillary tangles, further promoting microglial activation (Wang et al., 2023).

Astrocytes, another key cell type involved in neuroinflammation, also play a dual role in AD pathology. While they support neuronal health under normal conditions by maintaining the blood-brain barrier, regulating neurotransmitter levels, and providing metabolic support (Barnett et al., 2023), in AD, they become reactive. Activated astrocytes release inflammatory cytokines, chemokines, and ROS, further amplifying neuroinflammation and contributing to neuronal dysfunction (Lawrence et al., 2023). The interaction between activated astrocytes and microglia leads to a vicious cycle, where inflammation exacerbates the production of Aβ, tau phosphorylation, and neuronal loss (Goshi et al., 2020).

One of the key mechanisms through which neuroinflammation accelerates AD is the promotion of oxidative stress (Bai et al., 2022). Microglia and astrocytes generate ROS as part of their immune response, which, in excess, can damage neurons, synapses, and blood vessels (Kim et al., 2022; Ni et al., 2022). Oxidative stress further contributes to the disruption of neuronal signaling and synaptic plasticity, which are critical for cognitive function. Additionally, the inflammatory microenvironment associated with neuroinflammation increases the permeability of the BBB, allowing immune cells and pro-inflammatory molecules to infiltrate the brain, thus exacerbating the neurodegenerative process (Shen et al., 2024).

The interaction between neuroinflammation and other pathological features of AD is also critical. For example, the accumulation of Aβ plaques in the brain is not only a consequence of altered protein metabolism but also actively contributes to the neuroinflammatory response. Aβ peptides can directly activate microglia and astrocytes, leading to the release of pro-inflammatory cytokines, which in turn promote further Aβ aggregation (Hu J. et al., 2023). This forms a positive feedback loop that exacerbates both the neuroinflammation and the accumulation of toxic Aβ deposits, worsening disease progression. Similarly, tau pathology, another hallmark of AD, is also influenced by neuroinflammation. Activated microglia and astrocytes release pro-inflammatory mediators that can enhance tau phosphorylation, promoting the formation of neurofibrillary tangles and exacerbating neuronal death (Chen et al., 2023; Wang C. et al., 2021).

Given the critical role of neuroinflammation in AD, targeting the inflammatory processes in the brain represents a promising therapeutic strategy. Numerous studies have shown that modulating microglial and astrocytic activation can reduce neuroinflammation and attenuate AD pathology (Cai et al., 2022; Kummer et al., 2021). For instance, the use of anti-inflammatory agents, such as nonsteroidal anti-inflammatory drugs (NSAIDs) (McGeer et al., 2018) and other immune-modulating therapies (Mahdiabadi et al., 2022), has been explored for their potential to reduce neuroinflammation and slow disease progression. However, translating these findings into clinical success has been challenging, as many anti-inflammatory treatments fail to cross the blood-brain barrier effectively or have unwanted side effects (Xie et al., 2025).

In recent years, there has been growing interest in targeting neuroinflammation through more specific, localized approaches, such as the use of nanocarriers for drug delivery (Zhu et al., 2021). Nanocarriers offer the potential to directly target inflamed regions of the brain, delivering anti-inflammatory agents with greater precision and fewer systemic side effects. By enhancing the bioavailability and targeting specificity of therapeutic agents, nanocarriers could provide a more effective means of modulating neuroinflammation in AD.

## 3 Nanocarrier-based drug delivery: overcoming the blood-brain barrier and targeting neuroinflammation

**FIGURE 3:**
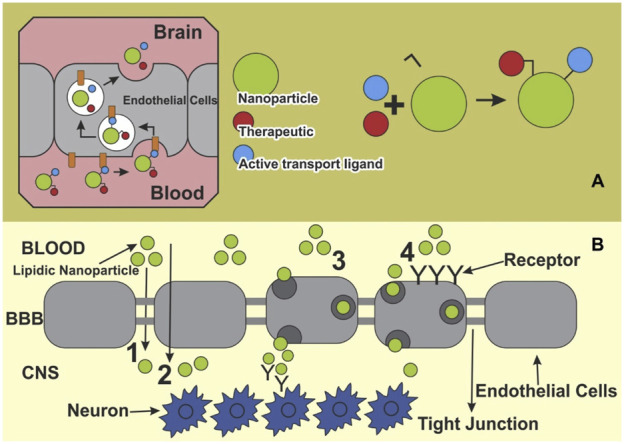
Main pathways for nano systems to cross blood-brain barrier. **(A)** Therapeutic ligands enabling active transport at the blood-brain barrier (BBB) can be conjugated to a nanoparticle, and then transported into the BBB. **(B)** A schematic presentation of various pathways of NPs crossing the BBB toward the brain: 1) Para cellular pathway, 2) transcellular pathway, 3) transcytosis, and 4) receptor mediated endocytosis. Extracted from author Saeedi et al. (2019). © 2018 Elsevier Masson SAS.

The BBB represents a formidable challenge in the treatment of CNS disorders, including AD. This selective permeability barrier protects the brain from harmful substances while maintaining homeostasis, but it also limits the effective delivery of therapeutic agents. For a drug to exert its effect in the brain, it must cross the BBB (Wu et al., 2023), which is composed of tightly packed endothelial cells, surrounded by a dense extracellular matrix and supported by astrocytes and pericytes. Conventional drug delivery strategies often fail to deliver adequate concentrations of therapeutic agents to the brain, necessitating innovative approaches to improve drug delivery to neuroinflammatory regions in AD. Nanotechnology, particularly the use of nanocarriers, has emerged as a promising strategy to overcome the challenges posed by the BBB and improve the delivery of drugs to inflamed regions of the brain. As depicted in Figure 3, NPs compounds pass through this barrier and reach neurons via different routes (Saeedi et al., 2019).

### 3.1 Properties of nanocarrier and their role in drug encapsulation

**FIGURE 4:**
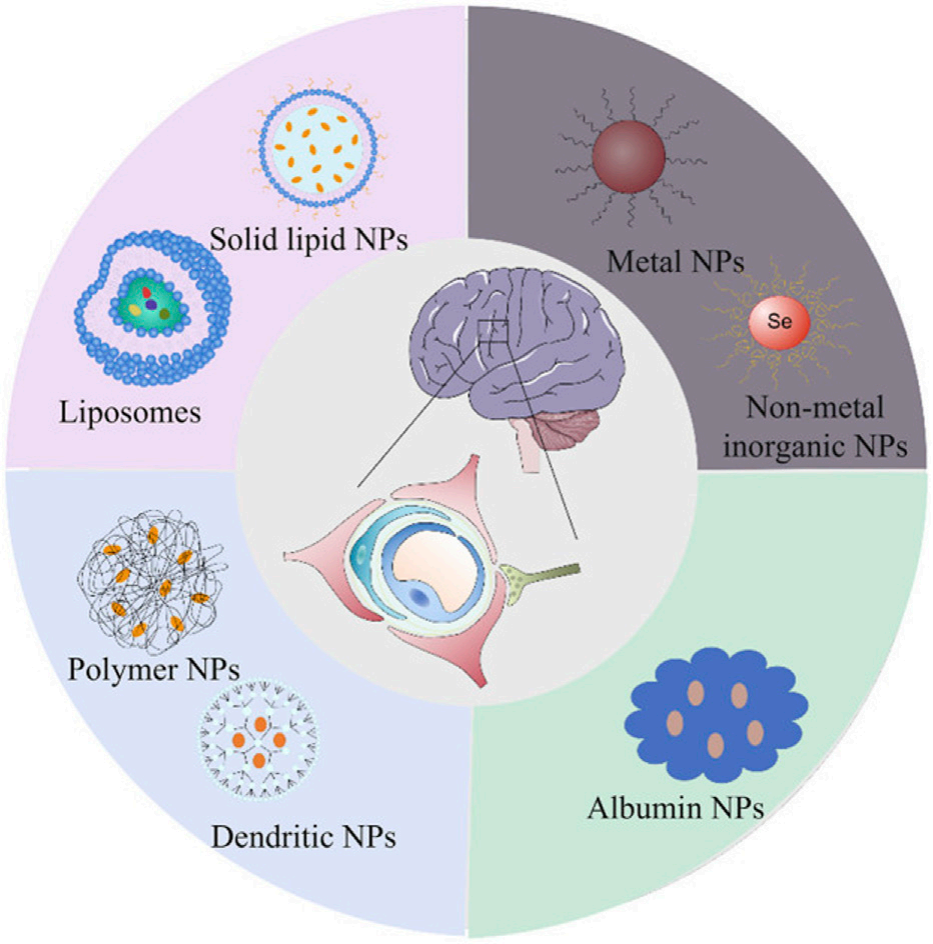
Schematic representation of the structures of the most commonly used nanomedicine types for Alzheimer’s disease: lipid (liposomes and solid lipid), organic (dendritic and polymer), inorganic (metal and non-metal inorganic) and biomimetic (albumin). Extracted from author Hu L. et al. (2023). Copyright © 2023 Hu, Tao, Jiang and Qin.

Nanocarriers are colloidal particles with a size typically ranging from 1 to 100 nm, and they can be composed of various materials, including lipids, polymers, and dendrimers (Kim et al., 2010; Satalkar et al., 2016). These advanced delivery systems offer significant advantages over traditional drug delivery methods, primarily due to their unique physicochemical properties and versatile functionalization capabilities. Figure 4 provides a schematic representation of the structures of the most commonly used nanomedicine types for AD (Hu et al., 2023b).

The exceptional utility of nanocarriers stems from several key characteristics. Their nanoscale dimensions facilitate penetration through biological barriers, most notably the BBB, while their versatile composition enables the encapsulation of a broad spectrum of therapeutic agents, encompassing hydrophobic drugs, proteins, nucleic acids, and small molecules (Patra et al., 2018; Wang J. et al., 2021). Among the most extensively investigated nanocarrier systems, liposomes, polymeric nanoparticles, and dendritic polymers have emerged as particularly promising platforms, each exhibiting distinct advantages in terms of drug stability, release kinetics, and biocompatibility (Nakamura et al., 2022; Beach et al., 2024; Dias et al., 2020).

A crucial aspect of nanocarrier technology lies in their capacity for therapeutic agent encapsulation, which serves multiple functions. Beyond providing protection against enzymatic degradation, encapsulation significantly enhances both drug stability and bioavailability (Bhattacharjee, 2022). Furthermore, the surface architecture of nanocarriers can be strategically modified through the incorporation of various functional moieties, such as polyethylene glycol (PEG) or specific antibodies (Kozma et al., 2020; Li et al., 2023). These modifications serve to enhance circulation time and enable targeted drug delivery. Of particular significance in the context of neurodegenerative disorders such as AD, surface modification techniques facilitate controlled drug release kinetics, enabling sustained therapeutic delivery. For example, the study by Meng et al. demonstrated that surface-modified Lf-TMC nanoparticles (NPs) exhibited enhanced brain targeting and prolonged residence time following intranasal administration, particularly in regions such as the olfactory bulb and hippocampus, resulting in increased drug accumulation. These findings suggest that Lf-TMC NPs can effectively improve the distribution of the drug in the brain, thereby enhancing therapeutic efficacy in AD treatment (Meng et al., 2018). Additionally, HupA-loaded Lf-TMC NPs displayed a sustained drug release profile, ensuring long-term therapeutic effects, which is crucial for the treatment of neurodegenerative disorders like AD.

### 3.2 Strategies for efficient blood-brain barrier penetration using nanocarriers

Current strategies for enhancing nanocarrier penetration across the BBB can be broadly categorized into three main approaches: passive targeting based on physicochemical properties, active targeting through specific ligand-receptor interactions, and receptor-mediated transcytosis. Among these, passive targeting represents the fundamental approach, wherein the inherent properties of nanocarriers are optimized to facilitate BBB penetration. Specifically, NPs under 100 nm with optimized surface properties, such as lipophilic coatings or PEGylation, have demonstrated the ability to diffuse across the BBB via adsorptive-mediated transcytosis or nonspecific interactions (Vargas et al., 2024). Lipid-based nanoparticles, including liposomes and solid lipid NPs (SLNs), are particularly effective due to their stability and ability to encapsulate both hydrophilic and hydrophobic drugs, making them suitable for targeting the brain (Fu et al., 2023). This is especially important in neurodegenerative diseases such as AD, where neuroinflammation leads to increased BBB permeability (Propson et al., 2021), allowing for greater nanoparticle accumulation in affected areas. In line with this, recent research has highlighted the effectiveness of nanogold-based therapies in tauopathies, particularly in AD. A study on tau P301L mutant mice demonstrated that nanogold conjugated with polyethylene glycol (Au-PEG) acted as a pseudo-nanochaperone, leading to a moderate improvement in learning ability and a reduction in phosphorylated tau burden (Vimal et al., 2020). Moreover, the treatment significantly decreased circulating tau levels, which can spread in a prion-like fashion, further supporting the potential of passive targeting approaches in neurodegenerative diseases. However, this strategy is inherently limited by factors such as the brain’s pathological state and the physicochemical properties of the nanocarriers, including their size, surface charge, and the nature of the coating (Xie et al., 2019).

Active targeting strategies represent a more sophisticated approach, focusing on enhancing nanocarrier specificity for the BBB (Bandyopadhyay et al., 2023). This methodology involves the surface modification of nanocarriers with specific ligands that interact with BBB endothelial cell receptors. Transferrin (Tf), a well-characterized glycoprotein that binds to transferrin receptors (TfR) on brain endothelial cells, has emerged as a prominent targeting moiety for facilitating nanoparticle BBB penetration (Kang et al., 2015). The study on Vitamin B12 (VB12)-loaded liposomes functionalized with Tf demonstrated that the dual-targeting system, leveraging Tf receptors on both the BBB and neuronal cells, significantly improved the delivery of VB12 to the brain (Andrade et al., 2022). The developed nanoparticles not only facilitated the sustained release of VB12 but also showed potential in delaying Aβ fibril formation. In another study, transferrin-conjugated nanoparticles (Tf-LioNs) successfully targeted amyloid plaques in the 5XFAD mouse model of AD (Choi et al., 2023). The Tf-functionalized melittin-loaded nanoparticles reduced amyloid plaque accumulation, especially in the hippocampus, suggesting that transferrin-based delivery systems could effectively target the brain and mitigate AD-associated pathology. Additionally, targeting strategies directed at the low-density lipoprotein receptor (LDLR) and insulin receptor (IR) have demonstrated significant potential for brain-targeted nanocarrier delivery. The mechanism of receptor-mediated transcytosis enables nanocarrier internalization by endothelial cells and subsequent transport across the BBB into the brain parenchyma, offering enhanced control and targeting precision. For example, Israel et al. (2023) research demonstrated that by conjugating microRNA (miRNA) and antisense RNA with a D-configured peptide, the Low-Density Lipoprotein Receptor-Related Protein-1 (LRP-1) transcytosis pathway could be effectively leveraged for targeted delivery across the BBB, achieving efficient and neuron-selective delivery in AD mouse models. Table 2 provides a comprehensive side-by-side comparison of the primary nanocarrier platforms discussed in this section, including liposomes, polymeric nanoparticles, and dendrimers.

**TABLE 2:** Comparison of nanocarrier platforms for drug delivery to the brain.

Nanocarrier platform	Drug encapsulation effectiveness	Clinical translation potential	BBB crossing ability	References
Liposomes	Liposomes provide moderate to high encapsulation effectiveness, especially for lipophilic and amphiphilic drugs. Their ability to encapsulate a wide variety of drugs is highly beneficial, though limitations exist for highly hydrophobic compounds	High clinical translation potential. Liposomes, especially PEGylated forms (e.g., liposomal doxorubicin), are already in clinical use. Liposomal formulations have been widely tested and used for CNS drug delivery	Liposomes can cross the BBB effectively, especially when surface-modified with PEG or other targeting ligands. Their ability to be modified for stealth properties aids in efficient BBB penetration	Zhou et al. (2018), Tenchov et al. (2023), Patel et al. (2018), Helm and Fricker (2015), Juhairiyah and de Lange (2021)
Polymeric Nanoparticles	High encapsulation efficiency for both hydrophilic and hydrophobic drugs. The polymeric matrix can encapsulate a wide range of drug types, improving drug stability and bioavailability in biological systems	Moderate to High potential. Polymeric nanoparticles show promise in preclinical studies and are entering clinical trials. However, there are challenges with scalability and ensuring consistent production	Polymeric nanoparticles, particularly those functionalized with PEG or other targeting ligands, have shown promising results in crossing the BBB, often via receptor-mediated endocytosis or active transport mechanisms	Zatorska-Płachta et al. (2021), Pagels and Prud’homme (2015), Ding et al. (2017), Raman et al. (2020)
Dendrimers	High encapsulation effectiveness, particularly for small molecules and nucleic acids, due to their highly branched structure that can accommodate a high amount of drug. Multifunctional surface groups allow for targeted delivery and high loading efficiency	Moderate clinical translation potential. Although dendrimers are highly efficient in drug delivery and gene therapy, concerns regarding toxicity and immunogenicity, as well as challenges in scaling up, limit their clinical applicability	Dendrimers have demonstrated excellent BBB penetration due to their small size and ability to facilitate endocytosis. Their high surface area and surface charge enable efficient interaction with the BBB, although toxicity concerns remain	Gorzkiewicz and Klajnert-Maculewicz (2017), Kharwade et al. (2022), Zhong et al. (2022), Zhu et al. (2019)

### 3.3 Nanocarrier drug release mechanisms and enhancing bioavailability in neuroinflammation

The effectiveness of nanocarrier-based drug delivery systems for treating neuroinflammation depends not only on their ability to penetrate the BBB but also on their capacity for controlled release and enhanced bioavailability. These attributes ensure that therapeutic agents reach their intended targets at the appropriate concentrations and over the required timeframes, thus effectively modulating neuroinflammatory processes (Qiu et al., 2021; Waheed et al., 2022).

One of the most critical factors in nanocarrier design for neuroinflammation is the ability to provide controlled and sustained drug release. Among these, stimuli-responsive systems that react to the specific conditions present in the neuroinflammatory microenvironment are of paramount importance. For example, pH-responsive nanocarriers take advantage of the mildly acidic environments often found in inflamed regions of the brain, particularly in AD (Chae et al., 2023). These carriers are typically made from polymers that undergo structural or chemical changes, such as protonation or hydrolysis, in response to the low pH, thus triggering the release of the encapsulated drug at the site of inflammation (Tang et al., 2015). This targeted approach minimizes systemic drug exposure and enhances therapeutic efficacy at the site of disease. Redox-responsive nanocarriers exploit the high levels of oxidative stress present in neuroinflammatory conditions. These carriers incorporate disulfide linkages or other redox-sensitive groups that break down in response to elevated ROS. For instance, Yuan et al. (Yuan et al., 2021) developed a ROS-responsive ruthenium nanoplatform (R@NGF-Se-Se-Ru) that enhances AD treatment by promoting neuronal regeneration and clearing Aβ deposits. Under near-infrared (NIR) irradiation, the nanoclusters inhibit Aβ aggregation and disaggregate Aβ fibrils. The diselenide bonds in the nanoclusters are broken in the high ROS environment of AD, leading to the release of small ruthenium nanoparticles. Additionally, the platform crosses the BBB using RVG peptides.

**FIGURE 5:**
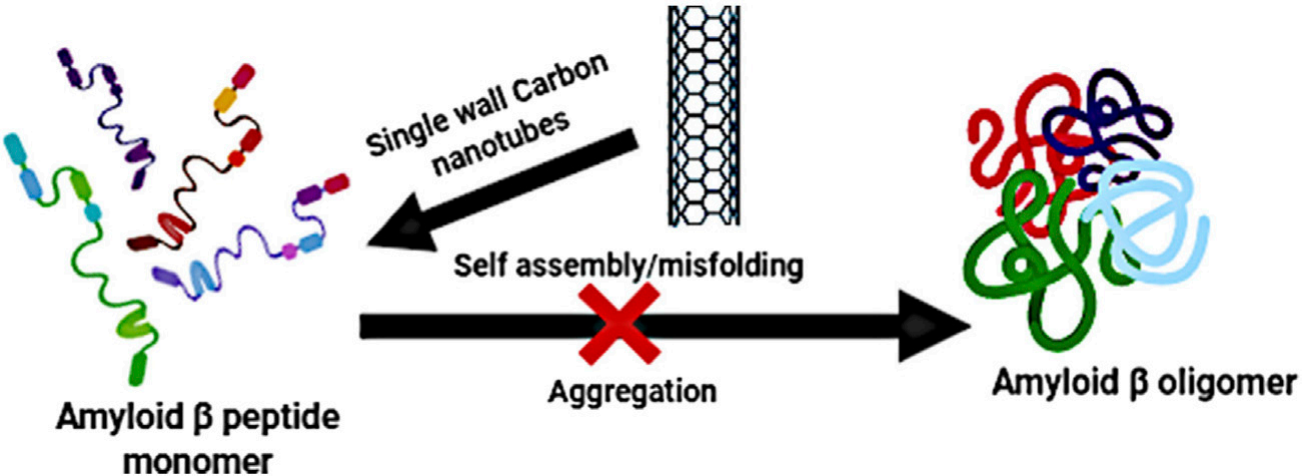
Using focused ultrasound to open the blood-brain barrier allows for the passage of drug molecules. Extracted from author Lipsman et al. (2018). © 2019 Elsevier B.V. All rights reserved.

Furthermore, externally regulated drug delivery systems represent another advanced approach in nanocarrier design for neuroinflammation, leveraging external stimuli such as ultrasound, magnetism, electrical signals, or irradiation to control drug release (Gopalan et al., 2020). These systems offer precise spatial and temporal control over drug delivery, making them highly suitable for treating conditions like AD. One notable external stimulus is ultrasound, which can be used to enhance drug delivery by temporarily disrupting BBB. Focused ultrasound, combined with microbubbles, can noninvasively open the BBB, allowing for the targeted delivery of drugs to the brain (Ogawa et al., 2022). This method has shown promise in preclinical models of AD, particularly in facilitating the clearance of Aβ. In a phase I safety trial, Lipsman et al. (2018) demonstrated that magnetic resonance-guided focused ultrasound, combined with intravenously injected microbubbles, can safely and reversibly open the blood-brain barrier in AD patients, enabling targeted drug delivery without significant adverse effects (Figure 5). Electrical signals can also be employed to modulate the release of drugs from nanocarriers. Conductive polymers or nanoparticles can be incorporated into drug delivery systems that respond to electrical stimulation (Gadhave et al., 2024). By applying external electrical fields, the drug release can be regulated to occur at precise moments, corresponding with the neural activity or specific stages of neuroinflammation. For example, Zhang et al. (Wu et al., 2015) developed an electrically responsive drug release platform, incorporating conducting polymer polypyrrole and graphene-mesoporous silica nanohybrids, which enables on-demand, spatial, and temporal control of drug delivery. This system effectively inhibits Aβ aggregation, reduces cellular ROS, and protects cells from Aβ-related toxicity, demonstrating the potential of electrical signals to modulate drug release for AD treatment.

In addition to controlled release mechanisms, enhancing the bioavailability of drugs is a critical aspect of nanocarrier-based drug delivery systems, particularly for neuroinflammation treatments (Deng et al., 2024). For instance, many drugs are prone to rapid degradation by plasma proteins or metabolic enzymes, which shortens their half-life and reduces their effective concentration at the target site. However, by encapsulating these drugs within nanocarriers, their stability is greatly improved, leading to prolonged circulation times and enhanced therapeutic efficacy (Huang et al., 2024). Moreover, nanocarriers can enhance the solubility of poorly soluble drugs, a frequent challenge in the development of treatments for neuroinflammatory diseases. For example, Curcumin, a hydrophobic polyphenol derived from the rhizomes of turmeric, is considered a potential therapeutic agent for AD due to its multifaceted effects on the nervous system (Hamaguchi et al., 2010). Despite its promising biological activities, the clinical application of curcumin is limited by its poor solubility, rapid clearance, and low stability (Maiti and Dunbar, 2018). In order to solve this problem, Huo et al. (2019) enhanced the solubility, stability, and bioavailability of curcumin by encapsulating it in selenium nanoparticles (Se-NP)-coated poly (lactic-co-glycolic acid) (PLGA) nanospheres. This formulation effectively targets amyloid plaques in AD, reducing Aβ load and improving memory function, offering a promising strategy for the treatment of AD. A summary of the main strategies used to enhance nanocarrier penetration across the BBB is provided in Table 3.

**TABLE 3:** Nanocarrier strategies for overcoming the blood-brain barrier in Alzheimer’s disease treatment.

Strategy	Mechanism	Nanocarrier types	Advantages	Limitations	References
Passive Targeting	Exploits nanocarrier properties (size, charge, hydrophobicity) to facilitate diffusion across the BBB	Liposomes, solid lipid nanoparticles (SLNs), polymeric nanoparticles	Non-invasive, does not require active modification	Limited efficiency, dependent on disease state and BBB integrity	Vargas et al. (2024), Fu et al. (2023), Propson et al. (2021)
Active Targeting	Utilizes ligand-receptor interactions to enhance nanocarrier uptake by BBB endothelial cells	Transferrin-functionalized nanoparticles, LDLR-targeted nanoparticles	Higher BBB penetration efficiency, increased specificity	Requires careful ligand selection, potential immunogenicity	Kang et al. (2015), Andrade et al. (2022), Choi et al. (2023)
Receptor-Mediated Transcytosis	Exploits natural receptor-mediated transport pathways for enhanced BBB crossing	Tf-functionalized liposomes, LRP-1 targeting nanoparticles	Efficient BBB transport, neuron-selective delivery	Potential competition with endogenous ligands, complex formulation requirements	Israel et al. (2023)
Stimuli-Responsive Nanocarriers	Release drugs in response to environmental triggers (pH, ROS, external stimuli)	pH-sensitive polymeric nanoparticles, ROS-responsive ruthenium nanoplatforms	Controlled drug release, minimizes systemic toxicity	Requires precise design and validation for specific conditions	Chae et al. (2023), Tang et al. (2015), Yuan et al. (2021)
Externally Regulated Delivery	Uses ultrasound, magnetic fields, or electrical signals to modulate drug release and enhance BBB permeability	Focused ultrasound with microbubbles, electrically responsive nanocarriers	Precise spatial and temporal control over drug delivery	Requires external equipment, may have limited applicability in clinical settings	Ogawa et al. (2022), Lipsman et al. (2018), Gadhave et al. (2024)

## 4 Experimental validation and preclinical applications

The development of nanocarrier-based drug delivery systems for the CNS requires a robust preclinical evaluation to ensure their efficacy, safety, and potential for clinical translation. This section explores the methodologies for assessing the application of nanocarriers in preclinical models, with a particular emphasis on AD. The following subsections highlight *in vitro* cellular models, *in vivo* animal models, neuroprotective effects, and behavioral assessments critical for evaluating the potential of nanocarriers in treating AD.

### 4.1 *In vitro* evaluation: cellular models

*In vitro* cellular models are essential for assessing the initial interactions between nanocarriers and target cells, providing valuable insights into the safety, cellular uptake, and therapeutic efficacy of nanocarrier-based drug delivery systems. Several cell types are utilized in these models, including neuronal cells, glial cells (microglia and astrocytes), and endothelial cells, all of which are implicated in the pathophysiology of AD.

#### 4.1.1 Neuronal cells and cytotoxicity evaluation

**FIGURE 6:**
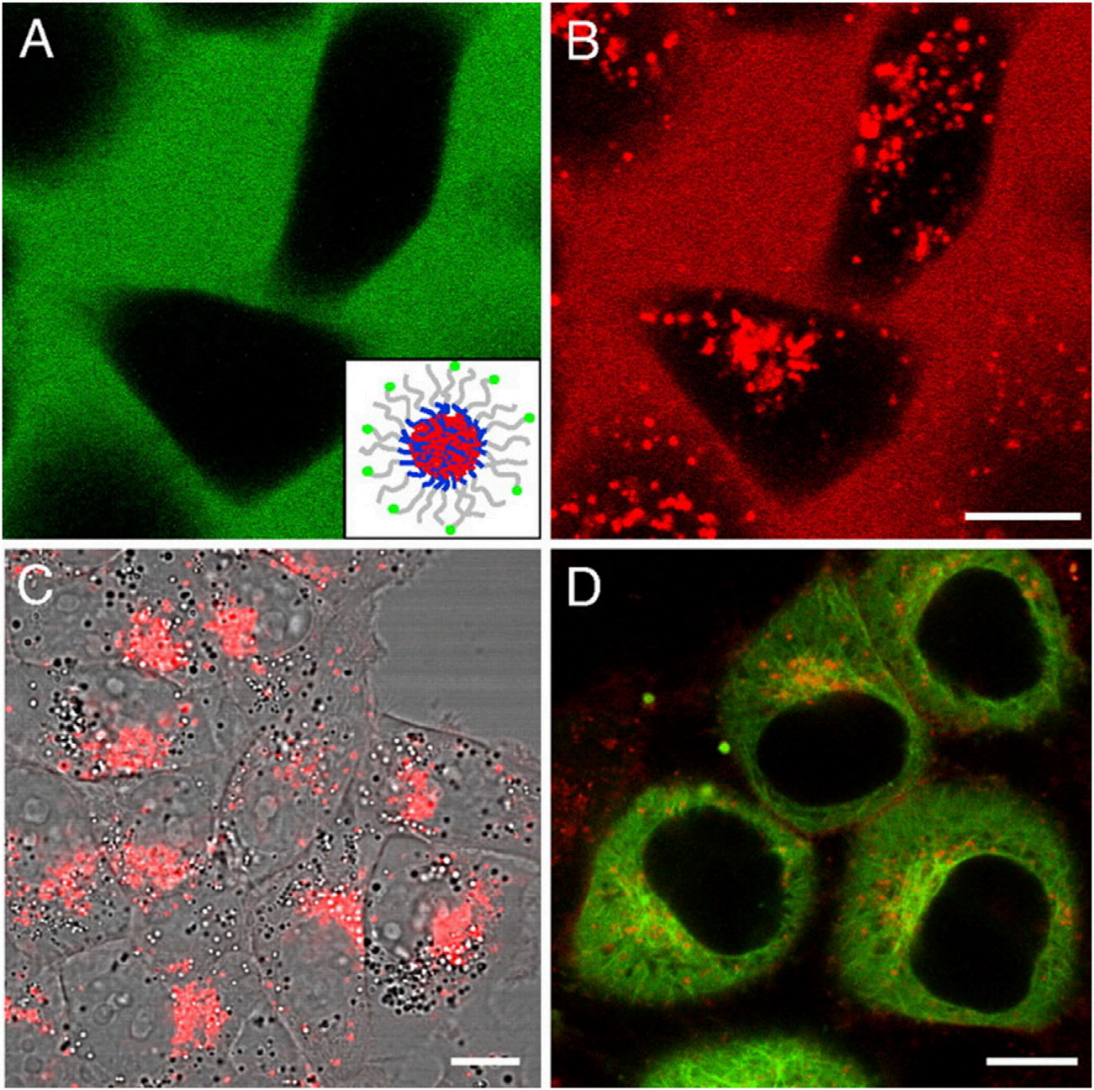
Cellular internalization of fluorescently labeled micelles and core-loaded hydrophobic probes inspected by confocal fluorescence microscopy. **(A,B)** KB cells treated with 0.2 mg/mL dual-labeled micelles for 24 h. The green color represents the FITC signal from labeled micelles, and the red color represents the DiI signal. (Inset) Diagram of a micelle labeled with FITC and loaded with DiI. **(C)** KB cells incubated with free DiI in DMSO at the final concentration of 0.1 μg/mL for 24 h. **(D)** HeLa cells incubated with 0.2 mg/mL micelles (physically loaded with 0.15% DiI) for 2 h. DiI was accumulated in the microtubule organizing center area. Extracted from author Chen et al. (2008). © 2008 by The National Academy of Sciences of the USA.

Neuronal cell lines, such as SH-SY5Y, as well as primary cortical neurons, are widely utilized in preclinical studies to evaluate the cytotoxicity and drug release profiles of nanocarriers, providing essential insights into their potential for CNS drug delivery (Pandey et al., 2024; Cepparulo et al., 2024). The use of these models allows for the assessment of both the safety and efficacy of nanocarrier systems in mimicking the complex environment of the brain. Given that successful nanocarrier-based therapies must not induce significant toxicity while ensuring effective drug delivery, cytotoxicity assays are among the first critical evaluations conducted. Commonly employed assays include the MTT (3-(4,5-dimethylthiazol-2-yl)-2,5-diphenyltetrazolium bromide) and resazurin assays, which measure cell viability following exposure to nanocarriers (Le Roux et al., 2017; Pourmadadi et al., 2023). These assays are particularly useful for quantifying metabolic activity, allowing researchers to determine whether the nanocarriers are causing any significant cellular damage. In the context of AD, minimizing toxicity is crucial to ensure that nanocarriers do not exacerbate neuronal dysfunction or contribute to additional cellular stress (Silva-Abreu et al., 2018). By assessing cell viability in these assays, researchers can establish a safe therapeutic window for the use of nanocarriers. To further understand the drug delivery potential of these nanocarriers, evaluating the internalization efficiency is essential. Cellular uptake studies provide valuable data on how effectively the nanocarriers are transported into the target cells and how well they can deliver their therapeutic payloads (Nelemans and Gurevich, 2020). Techniques such as fluorescence microscopy, flow cytometry, and confocal imaging are frequently employed for these purposes. Fluorescence-based methods allow researchers to track the fate of nanocarriers by labeling them with fluorescent markers, providing a visual confirmation of their uptake by neuronal cells. Additionally, flow cytometry enables quantitative analysis of uptake efficiency, assessing the percentage of cells that have internalized nanocarriers and the amount of carrier per cell (Nong et al., 2023). Confocal microscopy offers high-resolution imaging, allowing for the localization and visualization of nanocarrier distribution within the cells at a subcellular level, thus providing a more detailed understanding of how the nanocarriers interact with cellular structures such as endosomes or lysosomes. An example of this is shown in the figure depicting the cellular internalization of fluorescently labeled micelles in KB and HeLa cells, where fluorescence microscopy clearly demonstrates the uptake of dual-labeled micelles (FITC and DiI) and their accumulation in specific subcellular regions, such as the microtubule organizing center in HeLa cells (Chen et al., 2008). Figure 6 highlights the ability of fluorescence-based imaging to provide visual confirmation of nanocarrier internalization and offers a valuable example of how such techniques can be used to study the cellular distribution and behavior of nanocarriers. These cellular uptake studies are pivotal for establishing the drug delivery potential of nanocarrier systems. By evaluating both the internalization efficiency and the lack of significant cytotoxicity, researchers can identify the optimal formulations for advancing nanocarrier-based therapies. Moreover, these experiments provide insights into the behavior of the nanocarriers within the neuronal environment, including their ability to bypass cellular barriers and reach their intended targets, which is a crucial consideration in developing effective CNS drug delivery systems.

#### 4.1.2 Microglial and astrocytic interactions in neuroinflammation

In the context of AD, microglial activation contributes to the release of pro-inflammatory cytokines and other neurotoxic molecules, exacerbating neuronal damage and promoting disease progression. Therefore, evaluating the ability of nanocarrier systems to modulate glial cell activity and reduce neuroinflammation is critical in assessing their therapeutic potential for AD. Co-culture models that include both neurons and glial cells are essential for investigating the complex interplay between these cell types in AD (Keshavarz et al., 2024). Such models provide a more physiologically relevant environment, allowing researchers to simulate the inflammatory conditions observed in AD and evaluate how nanocarriers can influence this process. Specifically, the ability of nanocarriers to inhibit microglial activation (often characterized by morphological changes and the upregulation of pro-inflammatory cytokines) can be thoroughly assessed in these models (Huang et al., 2023). Cytokine assays, such as enzyme-linked immunosorbent assays (ELISA), are commonly used to quantify levels of key inflammatory markers, such as tumor necrosis factor-alpha (TNF-α), interleukin-1β (IL-1β), and interleukin-6 (IL-6) (Malpetti et al., 2025). These assays allow for a detailed analysis of how nanocarriers modulate the secretion of inflammatory cytokines in response to glial cell activation (Musumeci et al., 2022). Additionally, gene expression analysis using quantitative reverse transcription polymerase chain reaction (RT-PCR) provides valuable insights into the molecular mechanisms underlying the anti-inflammatory effects of nanocarriers. By measuring changes in the expression of specific inflammatory genes, researchers can assess the effectiveness of nanocarriers in shifting the glial cell response from a pro-inflammatory to a more neuroprotective phenotype (Abbas et al., 2021). These *in vitro* models and assays are critical for determining whether nanocarriers can successfully deliver anti-inflammatory drugs, such as corticosteroids, NSAIDs, or more novel compounds targeting specific inflammatory pathways, to the affected regions of the brain (Moreira et al., 2023).

### 4.2 *In vivo* evaluation: animal models of Alzheimer’s disease

Although *in vitro* models provide essential data on the fundamental interactions of nanocarriers, *in vivo* studies in animal models are indispensable for assessing the pharmacokinetics, biodistribution, and therapeutic effects of nanocarriers within a complex, living organism. Several transgenic mouse models of AD, such as the APP/PS1 or 5xFAD mouse models, are commonly used to evaluate the *in vivo* efficacy of nanocarriers.

#### 4.2.1 Pharmacokinetics and biodistribution

Understanding the pharmacokinetics and biodistribution of nanocarriers is essential for evaluating their efficacy and safety *in vivo*, especially when targeting the brain (Griffin et al., 2016). The ability to track the fate of nanocarriers in real-time allows researchers to obtain critical information regarding their systemic circulation, tissue penetration, and clearance mechanisms. To this end, advanced imaging techniques such as near-infrared fluorescence (NIRF) imaging and positron emission tomography (PET) are widely employed. These techniques enable dynamic monitoring of nanocarrier distribution across various tissues, including the brain, providing crucial insights into the pharmacokinetic behavior of nanocarriers under physiological conditions.

NIRF imaging is a particularly valuable tool for non-invasive, real-time tracking of nanocarriers in live animals (Kaku and Lim, 2021). By labeling the nanocarriers with near-infrared fluorescent dyes, researchers can monitor their distribution, identify target tissues, and assess the ability of these carriers to penetrate the BBB (Jing et al., 2021). NIRF imaging offers several advantages, including high sensitivity and deep tissue penetration, allowing for detailed visualization of nanocarrier movement and accumulation at various time points. This enables the evaluation of pharmacokinetic parameters, such as the half-life of the nanocarriers in the bloodstream and their clearance rates, which are critical for assessing both the therapeutic window and safety profile of the nanocarrier system (Chen et al., 2015).

Similarly, PET is another powerful imaging modality frequently used in preclinical pharmacokinetic studies (Pérez-Medina et al., 2020). PET imaging involves the use of radiolabeled nanocarriers, allowing for quantitative measurements of their distribution and accumulation *in vivo* (Lambidis et al., 2022). Unlike NIRF, PET imaging provides high-resolution, three-dimensional information on nanocarrier biodistribution, enabling more precise quantification of drug delivery to specific regions of the brain (Li et al., 2025). PET can also track the clearance of nanocarriers over time, offering insights into the pharmacodynamics of the drug, and helping to identify any potential off-target accumulation that may lead to adverse effects.

A comprehensive understanding of the pharmacokinetics of nanocarriers includes not only their ability to cross the BBB but also their ability to target and accumulate in the brain regions associated with AD. The ability to quantify drug concentrations in the brain over time provides essential information about the extent of drug delivery to the CNS, a critical factor in determining the therapeutic efficacy of nanocarrier-based drug delivery systems.

In addition to assessing drug distribution, the clearance of nanocarriers from the body is a key factor in evaluating their safety profile (Male and Gromnicova, 2022). Excessive accumulation of nanocarriers in non-target organs or prolonged circulation in the bloodstream may lead to undesirable toxicity (Ciappellano et al., 2016). Therefore, studies that track the clearance of nanocarriers through urinary, hepatic, or renal pathways are essential to ensure that these systems are both effective and biocompatible (Naumenko et al., 2019; Fan et al., 2022). Monitoring the metabolic fate of nanocarriers, including their degradation products, can also help in predicting the long-term safety and efficacy of the nanocarrier formulation in clinical applications.

#### 4.2.2 Behavioral assessments of cognitive and motor function

Behavioral assessments play a pivotal role in evaluating the therapeutic effects of nanocarriers on cognitive and motor functions in AD animal models (Puzzo et al., 2014). These tests provide valuable insight into the efficacy of drug delivery systems, particularly those utilizing nanocarriers, in reversing or mitigating the neurodegenerative processes characteristic of AD (K et al., 2021). Cognitive deficits in AD are primarily characterized by impairments in memory, learning, and executive function. As such, a variety of behavioral tests have been designed to assess these facets of cognition, with the Morris water maze (MWM) and Y-maze being two of the most widely employed techniques for evaluating cognitive performance in animal models (Sun et al., 2024).

The MWM is a widely accepted behavioral task used to assess spatial learning and memory (Bromley-Brits et al., 2011). In this test, animals are required to navigate a pool of water to locate a hidden platform using spatial cues. The test assesses the ability of the animal to learn the spatial location of the platform and retain this information over time. Cognitive dysfunction, as seen in AD, is typically reflected in an increased latency to find the platform, a reduced number of platform crossings, or a failure to retain the learned information across trials. In one study, lipoprotein conjugated polymeric nanoparticles were developed to enhance the clearance of Aβ fibrils, a hallmark of AD pathology, and reduce Aβ-induced neurotoxicity. When AD-induced rats were treated with these nanoparticles, the animals showed significant improvement in cognitive function, as measured by the MWM (Krishna et al., 2020). Specifically, the ApoE3-donepezil nanoparticles mitigated Aβ fibril formation and cognitive impairments, demonstrating their neuroprotective effects. The treated rats exhibited better performance in the MWM, with reduced latency to find the hidden platform, suggesting that the nanocarriers were effective in improving spatial learning and memory. Similarly, another study explored the use of nanoemulsions loaded with rivastigmine (RSG) for AD treatment (Roy et al., 2025). The optimal formulation of RSG nanoemulsion, with an average globular size of 202.3 nm, was tested in Long-Evans rats using the MWM to assess cognitive function. The results indicated that the nanoemulsion significantly reduced the travel distance of the rats in the maze, which is indicative of improved cognitive function. In addition, Qiao et al. (2022) demonstrated that in a D-galactose/aluminum chloride-induced AD model, probiotics enriched with selenium nanoparticles (L. casei ATCC 393-SeNPs) were evaluated for their effects on cognitive function using the MWM. The results showed that both the L. casei ATCC 393 and L. casei ATCC 393-SeNPs groups exhibited significant improvements in cognitive performance compared to the untreated controls. The rats treated with L. casei ATCC 393-SeNPs displayed an enhanced ability to locate the hidden platform in the MWM, indicating improved spatial memory. These studies collectively underscore the promising role of nanocarriers in improving cognitive performance in AD animal models.

In addition to cognitive decline, motor dysfunction is another critical feature of AD that significantly impairs the quality of life in patients. The deterioration of motor skills, including impaired coordination, balance, and overall motor control, is often seen in the later stages of the disease (Graff-Radford et al., 2021). The rotarod test is one of the most commonly used tools for assessing motor coordination, balance, and endurance in rodent models of AD (Fertan and Brown, 2022). The rotarod test measures an animal’s ability to maintain balance on a rotating rod (Shiotsuki et al., 2010). As the rod accelerates, animals are required to stay on it for as long as possible, with the time spent on the rod reflecting their motor coordination and balance. Motor impairments, such as those found in AD, are usually indicated by a reduced latency to fall or failure to maintain balance on the rod as the speed increases. The rotarod test is sensitive to motor dysfunction resulting from neurodegeneration in the central nervous system, particularly in regions such as the cerebellum and basal ganglia, which are involved in motor control. Following treatment with nanocarriers, improvement in performance on the rotarod test, such as an increased latency to fall or more consistent performance, indicates the restoration of motor function and coordination. For example, Vera et al. (2024) investigated the effects of a nanoparticle intranasal formulation, the Eagle Research Formulation of Ryanodex (ERFR), in both young adult and aged PS19 tau transgenic mice. The study aimed to assess the impact of ERFR treatment on cognitive and motor functions. Despite achieving higher concentrations of dantrolene in the brain than in the blood in aged mice, the results showed no significant improvement in motor performance, as assessed by the rotarod test, or in cognition and olfaction after chronic treatment with ERFR. This suggests that, although the intranasal ERFR formulation exhibited favorable pharmacokinetics, it did not significantly alleviate motor impairments in the PS19 mouse model of tauopathy. This study highlights the challenges in translating nanoparticle-based treatments into effective motor and cognitive improvements in neurodegenerative disease models, particularly those involving tau pathology.

Beyond the MWM, Y-maze, and rotarod tests, additional behavioral assessments are often used to provide a more comprehensive evaluation of the effects of nanocarrier-based treatments. For example, open-field tests can measure general locomotor activity, anxiety-like behavior, and exploratory drive (Kushawaha et al., 2024). These tests are useful for evaluating how treatments may impact emotional and motivational states, which can, in turn, influence performance in cognitive tasks. Furthermore, novel object recognition tests and fear conditioning paradigms can provide valuable information on more specific aspects of memory, such as recognition memory and associative learning (Çınar et al., 2022).

Together, these behavioral tests offer a multidimensional view of the therapeutic effects of nanocarriers in AD animal models. Not only do they assess improvements in cognitive and motor function, but they also provide insights into emotional and anxiety-related behaviors that can impact overall wellbeing. The integration of these diverse behavioral assays is essential for providing a comprehensive understanding of the efficacy of nanocarrier-based therapies in AD.

## 5 Challenges and future directions in nanocarrier-based therapy for Alzheimer’s disease

### 5.1 Current challenges and limitations

Despite the promising therapeutic potential of nanocarriers in AD, their clinical translation faces significant challenges. These barriers encompass issues related to manufacturing complexities, safety concerns, biological hurdles, and the growing need for personalized treatments. This section provides a detailed exploration of these challenges, while offering potential solutions and future directions to overcome these obstacles.

#### 5.1.1 Long-term safety concerns: immunogenicity and neurotoxicity

A major concern regarding nanocarrier-based therapeutics is their long-term safety, particularly concerning immunogenicity and neurotoxicity. When administered repeatedly, nanocarriers may provoke immune responses that result in the production of antibodies, potentially accelerating their clearance from the body and diminishing their therapeutic efficacy (Comberlato et al., 2019). Furthermore, repeated exposure to nanocarriers could lead to neurotoxicity, which is of particular concern in treatments targeting the CNS, including the brain (Szabat-Iriaka and Le Borgne, 2021).

To address these safety concerns, several preclinical safety evaluation strategies should be implemented (Szabat-Iriaka and Le Borgne, 2021). Long-term toxicological studies in animal models are essential to assess the potential for immune activation and neuroinflammation resulting from chronic nanocarrier exposure (Lu et al., 2023). These studies should incorporate methods such as cytokine profiling, histopathological analysis, and monitoring of cognitive function over extended periods. Furthermore, biocompatible nanocarriers, such as those modified with PEG, may reduce immune activation, enhancing circulation time and lowering the risk of immune system clearance (Tenchov et al., 2023). Additionally, neurotoxicological studies should evaluate whether repeated administration leads to cumulative effects on neurodegeneration or cognitive decline. Key biomarkers of neuroinflammation, including TNF-α, IL-6, and other cytokines, should be closely monitored to assess potential neurotoxic impacts.

#### 5.1.2 *In vivo* degradation, clearance, and accumulation

The degradation, clearance, and potential accumulation of nanocarriers in the brain and peripheral tissues represent significant challenges in their clinical application (Wang et al., 2025). Nanocarriers are designed to cross the BBB and deliver therapeutic agents to the CNS, but their accumulation in non-target tissues or the brain could lead to toxicity or diminish their therapeutic efficacy (Blanco et al., 2015; Zelepukin et al., 2024). Furthermore, the process by which nanocarriers are cleared from the brain after fulfilling their therapeutic role is not well understood, complicating their use for chronic treatments.

To address these concerns, biodegradable nanocarriers offer an ideal solution to mitigate long-term accumulation. Nanocarriers made from biodegradable polymers such as PLGA or polylactide can degrade into non-toxic by-products, which are eliminated through natural metabolic processes (Garrigós et al., 2024). Additionally, the use of enzymatically responsive materials that degrade when exposed to specific enzymes could accelerate clearance, reducing the risk of prolonged accumulation in the brain and peripheral tissues (Xie et al., 2023).

The clearance of nanocarriers should be closely monitored through real-time imaging techniques, such as MRI or fluorescence imaging, in preclinical models (Porret et al., 2023). These techniques enable tracking of nanocarrier distribution, allowing assessment of whether they accumulate in non-target tissues like the liver or kidneys, or remain in the brain beyond the desired therapeutic time window. Regular evaluations of biological clearance, including urinary excretion and hepatic metabolism, are crucial for understanding the elimination pathways of nanocarriers and ensuring their safety during chronic administration.

#### 5.1.3 Biomarker-driven patient classification for personalized nanocarrier treatments

AD is a highly heterogeneous condition, with variability in disease progression and underlying biological mechanisms among individuals. To optimize the effectiveness of nanocarrier-based therapies, it is essential to tailor treatments based on the specific characteristics of individual patients. Biomarker-driven patient classification is key to this process, enabling the personalization of therapy to meet the unique needs of each patient.

For patient stratification, genetic biomarkers, such as mutations in the APP or PSEN1 genes, can identify individuals with familial forms of AD who might benefit from gene therapy delivered via nanocarriers (Nikolac Perkovic et al., 2021). In addition, neuroimaging techniques, including PET scans, can provide crucial insights into the distribution of amyloid plaques and tau tangles, guiding the use of nanocarriers that target these specific aggregates (Graff-Radford et al., 2021). Moreover, neuroinflammatory biomarkers like C-reactive protein (CRP) or TNF-α can indicate the presence of neuroinflammation, suggesting that patients with elevated levels of these biomarkers may benefit from nanocarriers targeting neuroinflammation (Carter et al., 2019; Lista et al., 2024).

We recommend incorporating these biomarkers into clinical trial designs for nanocarrier-based therapies to enable better patient selection. Tailored nanocarrier formulations can then be designed to specifically address the pathophysiological mechanisms unique to each patient’s condition, improving therapeutic outcomes and minimizing adverse effects.

#### 5.1.4 Off-target effects, biodistribution variability, and non-specific drug release

Concerns regarding off-target effects, biodistribution variability, and non-specific drug release are major hurdles in the development of nanocarrier-based therapies. Although targeted delivery is one of the primary advantages of nanocarriers, ensuring precise targeting to specific tissues or cells remains a challenge. Biodistribution variability can lead to unpredictable drug delivery, potentially reducing the efficacy of the treatment and increasing the risk of toxicity (Applegate et al., 2022).

To address these concerns, we propose advanced targeting strategies, including the use of dual-targeting ligands and stimuli-responsive systems to enhance specificity and improve the control over drug release. For example, targeting ligands such as transferrin (for transferrin receptor targeting) (Choudhury et al., 2018) or vitamin B12 (for targeting both transferrin receptors and neuronal cells) (Liu et al., 2019a) can enhance the accuracy of nanocarriers in reaching amyloid plaques or tau tangles in the brain. Additionally, stimuli-responsive nanocarriers that release their therapeutic payloads in response to specific triggers (e.g., changes in pH, temperature, or enzymatic activity) can reduce non-specific drug release, ensuring that the drug is only released at the target site (Das et al., 2020). Furthermore, computational modeling can be used to predict the biodistribution of nanocarriers, optimizing factors such as size, surface charge, and drug-loading capacity to reduce the risk of non-specific interactions and enhance targeted drug delivery.

### 5.2 Future research directions

The future of nanocarrier-based drug delivery systems for AD holds great promise. However, to fully realize their potential, multiple research avenues must be pursued to overcome existing challenges. In this section, we present detailed, actionable research directions, each targeting a critical area in the development of these systems.

#### 5.2.1 Development of theranostic nanocarriers for Alzheimer’s disease

The integration of therapeutic and diagnostic functions into a single nanocarrier, known as theranostic nanocarriers, has been successfully demonstrated in various medical fields, including organ transplantation (Guo et al., 2012) and cancer treatment (Liu et al., 2019b), where nanocarriers deliver drugs while enabling real-time monitoring through imaging. Translating this dual-function approach to AD presents a promising opportunity for improving both treatment efficacy and monitoring capabilities in a non-invasive manner.

In the context of AD, the potential to develop theranostic nanocarriers could involve adapting successful strategies from other fields where this approach has been proven. For example, superparamagnetic iron oxide nanoparticles (SPIONs) and quantum dots have been widely used in organ transplantation to enable the tracking of tissue grafts through magnetic resonance imaging (MRI) and fluorescence, respectively (Aghighi et al., 2018; Mannucci et al., 2017). These materials can also be functionalized for targeting amyloid plaques and tau aggregates in AD. In cancer, nanocarriers combined with imaging agents like MRI contrast agents or fluorescence markers have been successfully used to track drug delivery to tumor sites (Wei et al., 2023). This concept could be directly adapted to AD, where the therapeutic goal is to clear amyloid plaques or prevent tau neurofibrillary tangles.

For instance, SPIONs could be functionalized with anti-amyloid antibodies or anti-tau antibodies, allowing the nanocarriers to target amyloid plaques or tau tangles while also serving as contrast agents for MRI scans. Similarly, quantum dots, which have been used for tracking in cancer therapy, could be employed for fluorescence-based imaging of the brain, enabling the tracking of drug release and distribution in real-time. The use of multimodal imaging (Liang et al., 2023), such as combining MRI and fluorescence, could provide deeper insights into the spatial distribution of nanocarriers in the brain, potentially highlighting areas of amyloid plaque accumulation or tau pathology.

Building on these existing methodologies, AD-specific theranostic nanocarriers could be designed to deliver targeted therapies, such as small molecules, peptides, or biologics, while simultaneously monitoring their distribution, targeting efficiency, and therapeutic effects. For example, using SPIONs in combination with PET could enable the precise monitoring of the deposition of amyloid plaques and the subsequent clearance following drug treatment, thus providing a real-time feedback mechanism for clinicians and researchers.

#### 5.2.2 Standardized methodologies for *in vitro* and *in vivo* evaluation

The reproducibility and comparability of nanocarrier systems across studies are critical for advancing Alzheimer’s research. To improve the standardization of evaluation methods, we must establish common protocols for both *in vitro* and *in vivo* assessments. Specifically, there is a need for standardized 3D models of the BBB to evaluate nanocarrier penetration and targeting (Pavlou et al., 2025). Existing 2D cell cultures often fail to replicate the complexity of the BBB, and 3D models can better simulate the physiological conditions of AD. Additionally, establishing universal standards for the *in vivo* models used in AD studies is necessary. A consistent transgenic mouse model, such as the APP/PS1 mouse (Kosel et al., 2020), which exhibits amyloid plaque formation, should be adopted in all preclinical studies.

Moreover, multi-center collaborations will be essential to ensure the consistency and reliability of results. By pooling resources and data from multiple laboratories, we can minimize variability and ensure that findings from individual studies can be generalized. Additionally, adopting international standards like those set by ISO or ASTM for the characterization and testing of nanocarriers would ensure that results are comparable across studies and laboratories.

#### 5.2.3 Artificial intelligence and machine learning in nanocarrier design

The application of artificial intelligence (AI) and machine learning (ML) in the design of nanocarriers holds transformative potential. Generative adversarial networks (GANs), a form of AI, can be used to design new nanomaterials by predicting the optimal structural and physicochemical properties for efficient drug delivery (Mazumdar et al., 2025; Alshawwa et al., 2022). ML algorithms can be used to predict the interactions between nanocarriers and biological barriers, such as the BBB, by analyzing vast datasets on material properties, biological responses, and patient-specific data (Gao et al., 2024).

Furthermore, AI can be utilized to optimize personalized dosing regimens (Ribba et al., 2020). By analyzing genomic data, such as the patient’s genetic predispositions to AD or metabolic pathways, AI can predict how a given patient might respond to a particular nanocarrier formulation. This approach will allow for the development of more precise and individualized treatment plans, reducing side effects and enhancing treatment efficacy. Additionally, integrating molecular dynamics simulations with AI can predict how nanocarriers will interact with cellular membranes, ensuring more accurate and safer drug delivery systems (Tang, 2023).

#### 5.2.4 Synergistic integration with other therapeutic modalities

Combining nanocarriers with other therapeutic modalities, such as gene therapy and immunotherapy, offers a highly promising, multifaceted approach to treating AD. This integration can address the complex pathophysiology of AD by targeting multiple biological pathways simultaneously, resulting in more effective treatments with reduced side effects.

One of the most promising applications of nanocarriers in AD is their use in gene therapy. For example, CRISPR/Cas9 technology has revolutionized the ability to edit specific genes with high precision (Bhardwaj et al., 2022). Nanocarriers can be engineered to deliver CRISPR components (such as Cas9 and guide RNA) directly to neurons, facilitating the correction of genetic mutations that drive AD pathology (Joshi et al., 2022). Mutations in the APP gene and PSEN1 gene, which are known to cause early-onset familial AD, could be corrected using this approach. Furthermore, RNA interference (RNAi) represents a powerful gene-silencing strategy for AD, particularly for downregulating the expression of genes implicated in Aβ production and tau pathology. Among RNAi agents, small interfering RNA (siRNA) has garnered increasing attention due to its ability to selectively suppress disease-associated targets at the post-transcriptional level. However, the therapeutic efficacy of siRNA relies heavily on the efficiency of its delivery, as naked siRNA is highly susceptible to enzymatic degradation and exhibits poor cellular uptake. To overcome these challenges, LNPs have been developed as promising carriers for siRNA delivery (Ahmed, 2024). These nanoparticles not only protect siRNA molecules from nuclease-mediated degradation but also enhance their uptake by neuronal cells, enabling gene silencing within the brain. For example, Wang et al. (2018) developed PEGylated poly (2-(N,N-dimethylamino) ethyl methacrylate)-based nanocomplexes, surface-modified with the CGN peptide to facilitate BBB penetration and the Tet1 peptide to achieve neuron-specific targeting. Their system, termed CT/siRNA, successfully delivered siRNA targeting BACE1, a critical enzyme in Aβ generation, resulting in over 50% gene knockdown in neurons. In APP/PS1 transgenic mice, this nanoplatform effectively reduced amyloid plaque burden, decreased tau phosphorylation, and restored synaptic function and cognitive performance to levels comparable to wild-type controls.

Another promising combination is the use of immunotherapy with nanocarriers. Immunotherapy has gained significant attention due to its ability to target amyloid plaques and tau tangles, two pathological features of AD (Jucker and Walker, 2023). Recent studies have explored the use of monoclonal antibodies like Aducanumab and Lecanemab, which target amyloid plaques, as well as anti-tau antibodies that target tau tangles. However, delivering these large molecules across the BBB remains a significant challenge. Nanocarriers offer a solution by encapsulating therapeutic antibodies and ensuring their targeted delivery to the brain. For example, PepH3, a cationic peptide derived from the Dengue virus type-2 capsid protein, has been conjugated to nanocarriers to enhance BBB penetration. In preclinical models, PepH3 significantly increased the uptake of NPs into brain endothelial cells and facilitated transcytosis across both rat and human BBB models. This approach enables the efficient delivery of single-domain antibodies (sdAb) recognizing Aβ oligomers, providing a targeted method to treat AD while potentially reducing side effects associated with systemic antibody delivery (Szecskó et al., 2025). Similarly, PLGA-based nanoparticles functionalized with anti-transferrin receptor monoclonal antibodies (OX26) and anti-Aβ antibodies (DE2B4) have been used to improve the brain delivery of peptide-based drugs, such as iAβ5, which inhibits Aβ aggregation (Loureiro et al., 2016). This nanocarrier system significantly enhanced the uptake and controlled delivery of iAβ5 across the BBB in porcine brain capillary endothelial cell models, demonstrating the potential of immunonanoparticles for targeted drug delivery in AD.

#### 5.2.5 Regulatory challenges and recommendations

The translation of nanocarrier-based therapies from preclinical models to clinical application faces significant regulatory hurdles. One of the primary challenges is the lack of a clear and consistent regulatory framework for nanomedicines. To address this, we recommend the establishment of a classification system for nanocarriers, which takes into account their unique physicochemical properties, such as size, surface charge, and biodegradability. This system would allow regulators to assess nanocarriers based on their specific characteristics rather than applying a one-size-fits-all approach.

Additionally, the adoption of *in vitro* alternative testing models and biomarker-based patient selection can streamline the approval process. Regulatory agencies should also facilitate the use of real-world data to support drug approval, which would allow faster evaluation of nanocarrier therapies, particularly in complex diseases like AD. The International Conference on Harmonisation (ICH) guidelines could serve as a valuable framework for harmonizing regulatory practices across regions, ensuring the faster and more efficient approval of nanomedicines.

#### 5.2.6 Overcoming translational barriers

The translation of nanocarrier-based therapies into clinical practice faces several translational barriers, including issues related to scalable manufacturing, economic feasibility, and patient compliance. One promising approach to overcoming these challenges is the use of continuous manufacturing techniques, such as microfluidic synthesis, which allow for the production of nanocarriers at a large scale while maintaining precise control over their size and properties. This approach could drastically reduce production costs and increase the availability of nanocarrier-based treatments. To improve patient compliance, the development of long-acting formulations that reduce the frequency of administration is crucial. Nanocarriers with sustained-release capabilities could be engineered to slowly release therapeutic agents over weeks or months, thereby improving patient adherence to treatment regimens. Furthermore, wearable devices that monitor drug release and patient responses could provide real-time data, allowing for personalized treatment adjustments. Additionally, clinical trial design should evolve to incorporate adaptive trial designs and biomarker-enriched strategies to identify the most appropriate patient populations for nanocarrier-based therapies. This approach would enable faster, more efficient clinical trials and improve the likelihood of successful outcomes in diverse patient groups.

## 6 Conclusion

Nanocarrier-based drug delivery systems represent a transformative approach in the treatment of AD, particularly in targeting neuroinflammation, overcoming the BBB, and enhancing drug bioavailability. Through advancements in nanotechnology, various types of nanocarriers, including lipid-based nanoparticles, polymeric carriers, and dendrimers, have demonstrated promising capabilities in delivering therapeutic agents to inflamed neural tissues with improved precision and efficacy. Preclinical studies have provided substantial evidence that these systems can mitigate neuroinflammatory responses, reduce Aβ and tau pathology, and improve cognitive functions in animal models.

Despite these encouraging findings, several challenges remain that hinder the clinical translation of nanocarrier technologies. Large-scale manufacturing of nanocarriers presents issues related to production cost, reproducibility, and quality control, while regulatory frameworks for nanomedicine require further refinement to accommodate the unique properties of these delivery systems. Additionally, biological challenges such as long-term safety, potential immunogenic responses, and species-specific differences in BBB permeability complicate the transition from preclinical models to human applications.

Future research should focus on optimizing nanocarrier formulations for enhanced biocompatibility and targeted delivery, incorporating emerging technologies such as gene therapy, immunotherapy, and AI-driven drug design. Furthermore, interdisciplinary collaboration among material scientists, pharmacologists, and clinicians will be critical in developing scalable and clinically viable nanocarrier-based therapies. By addressing these challenges, nanocarriers have the potential to revolutionize AD treatment strategies, offering a more effective and personalized approach to combating this devastating neurodegenerative disorder.
